# Crystal structure of the CD33/Fab-10C8 complex elucidates the mechanism of antibody antagonism in HBV-induced immunosuppression

**DOI:** 10.1186/s12929-026-01248-9

**Published:** 2026-05-29

**Authors:** Yi-Hung Yeh, Min-Guan Lin, Pei-Shan Sung, Shie-Liang Hsieh, Chwan-Deng Hsiao

**Affiliations:** 1 Institute of Molecular Biology, Academia Sinica, Taipei, 115 Taiwan; 2 School of Pharmacy, College of Pharmacy, Taipei Medical University, Taipei, 110 Taiwan; 3 Immunology Research Center, National Health Research Institutes, Zhunan, 350 Taiwan; 4 Institute of Clinical Medicine, National Yang Ming Chiao Tung University, Taipei, Taiwan; 5 Cancer and Immunology Research Center, National Yang Ming Chiao Tung University, Taipei, Taiwan

**Keywords:** CD33, Fab-10C8, Immunosuppression, Hepatitis B virus (HBV), Inhibitory Siglec, SHP-1/SHP-2 signaling

## Abstract

**Background:**

Hepatitis B virus (HBV) infection persists through immune evasion strategies, including engagement of the inhibitory receptor CD33 (Siglec-3) by α2,6-linked sialoglycans on HBsAg. This interaction induces ITIM phosphorylation and SHP-1/2 recruitment, dampening myeloid cell activation. The monoclonal antibody 10C8 has been identified as a potent antagonist of CD33, but the structural basis for its inhibitory activity remains unclear.

**Methods:**

We determined the 3.2 Å crystal structure of the human CD33 extracellular domain (CD33-ECD) in complex with the Fab fragment of 10C8 (Fab-10C8). The stoichiometry and assembly of the complex in solution were validated by size-exclusion chromatography and analytical ultracentrifugation (AUC). Structural comparisons with apo CD33 were performed to assess potential Fab-induced conformational changes, and buried surface area analyses were conducted to characterize binding interfaces.

**Results:**

The structure reveals a 2:2 stoichiometry complex comprising two CD33-ECD molecules and two Fab-10C8 fragments, consistent with the 157 kDa molecular mass determined by AUC. Each Fab engages the V-set domain through extensive complementarity-determining region (CDR) interactions, burying ~612 Å^2^ of surface area, while inter-Fab contacts stabilize a compact dimeric arrangement of CD33-ECD. This geometry differs markedly from the relaxed apo state, showing a ~21° reduction in the dimer angle. The antibody-binding epitope is adjacent to, but not overlapping with, the canonical sialic acid-binding cleft, leading to steric occlusion that prevents HBsAg engagement. Together with a potential Fab-induced ectodomain compaction, this conformation restricts CD33 clustering and likely precludes SHP-1/2 recruitment, providing a mechanistic explanation for the antagonistic activity of 10C8.

**Conclusions:**

This study provides the structural insight into antibody-mediated inhibition of CD33. By locking CD33 into a sterically occluded, signaling-refractory conformation, 10C8 effectively reverses HBV-induced immunosuppression, thereby restoring host antiviral activity. These findings establish a structural framework for rational design of Siglec-targeted immunotherapies against chronic viral infections and other immune regulatory disorders.

**Supplementary Information:**

The online version contains supplementary material available at 10.1186/s12929-026-01248-9.

## Introduction

Hepatitis B virus (HBV) infection remains a major global health concern, frequently progressing to liver cirrhosis and hepatocellular carcinoma (HCC) in patients with chronic HBV (CHB) infection [1–3]. CHB is characterized by four distinct clinical phases, each defined by varying degrees of hepatic inflammation, viral replication, and immune response [1–3]. While CHB patients generate antibodies against hepatitis B virus core antigen (HBcAg) and hepatitis B virus envelope antigen (HBeAg), they consistently fail to produce anti-HBsAg (hepatitis B virus surface antigen) antibodies across all phases of infection [1–3]. This phenomenon is closely associated with the persistent presence of HBsAg and HBV DNA in the serum, both of which are sustained by the activity of covalently closed circular DNA (cccDNA) within infected hepatocytes [4].

Although the ideal therapeutic goal is to eliminate HBsAg, induce anti-HBsAg antibodies, and eradicate cccDNA [4], currently approved antiviral therapies including nucleotide analogues, immune checkpoint inhibitors, and therapeutic vaccines have limited efficacy in achieving functional cure. Only a minority of patients exhibit sustained reductions in HBsAg following treatment with nucleotide analogues [5–7], anti-PD-1 monoclonal antibodies (mAbs) [8], or therapeutic HBV vaccines [9]. Notably, immune checkpoint blockade targeting the PD-1/PD-L1 pathway may reactivate HBV in cancer patients with CHB [10], highlighting the limitations of nonspecific T cell-mediated immune activation as a treatment strategy. These findings underscore the urgent need to explore alternative immunotherapeutic targets for CHB.

One potential target is the hepatitis B virus surface antigen (HBsAg), which carries a biantennary α2,6-linked sialoglycan at Asn-146 that is structurally identical to endogenous human α2,6-linked sialoglycans [11–14]. Sialoglycans are known to modulate immune responses by binding to sialic acid-binding immunoglobulin-like lectins (SIGLECs) [15], a family of immunoinhibitory receptors. These receptors contain cytoplasmic immunoreceptor tyrosine-based inhibitory motifs (ITIMs) that recruit SHP-1 and SHP-2 phosphatases to attenuate cellular activation [16, 17]. The signaling mechanism of inhibitory SIGLECs mirrors that of other ITIM-containing checkpoint receptors such as PD-1 and CTLA-4 [18–20], which have been successfully targeted by antagonistic mAbs in cancer immunotherapy [21–24].

Polymorphisms in SIGLEC-encoding genes have been implicated in multiple human diseases, further supporting their immunoregulatory importance. For example, SNPs in *SIGLEC3* (CD33) have been associated with Alzheimer’s disease [25], while variants in *SIGLEC9* and *SIGLEC8* are linked to chronic obstructive pulmonary disease (COPD) [26] and bronchial asthma [27], respectively. These observations suggest that SIGLECs play critical roles in the pathogenesis of diverse inflammatory and immune-related conditions.

CD33, also known as SIGLEC-3, is a transmembrane receptor primarily expressed on cells of the myeloid lineage, including monocytes, macrophages, and myeloid-derived suppressor cells. Structurally, CD33 is a single-pass type I membrane protein consisting of an extracellular region (ECD) with two immunoglobulin-like domains (V-set and C2-set), a transmembrane α-helix, and a cytoplasmic tail harboring two conserved ITIMs [16, 17]. The N-terminal V-set domain mediates sialic acid recognition, while the C2-set domain may stabilize ligand binding and facilitate protein–protein interactions. Ligand engagement induces phosphorylation of the ITIM motifs, recruiting SHP-1 and SHP-2 phosphatases to initiate downstream inhibitory signaling cascades involved in cell proliferation, differentiation, and immune suppression [16, 17]. CD33 is also subject to *N*-linked glycosylation, which may influence its ligand affinity, cellular trafficking, and stability [18–20]. The N-terminal V-set domain mediates sialic acid recognition through a binding site centered on the conserved Arg119, which forms a salt bridge with the sialic acid carboxylate [28]. This interaction is further stabilized by an aromatic cluster (Phe21, His45, and Tyr127) [28] and the STKYSYK motif (residues 124–130) [29], which collectively provide the structural basis for the receptor’s preference for α2,6-linked sialoglycans [28, 29].

Recent studies have identified CD33 as an immune checkpoint receptor exploited by HBV to suppress host immunity [29]. Specifically, the α2,6-biantennary sialoglycans on HBsAg bind directly to the sialic acid recognition domain of CD33 [29], triggering downstream phosphorylation of CD33 ITIMs and recruitment of SHP-1/-2, thereby promoting immune tolerance [29]. In our previous study, we demonstrated that a CD33-blocking monoclonal antibody, 10C8, disrupts this interaction and restores cytokine production in peripheral blood mononuclear cells (PBMCs) derived from CHB patients [29].

To better understand the molecular basis of this therapeutic mechanism, we aim to determine the crystal structure of the CD33/10C8 complex. Structural elucidation will reveal how 10C8 competes with HBV for CD33 binding and sterically hinders HBsAg from re-engaging the receptor. These insights could facilitate the rational design of improved anti-CD33 antibodies and support the development of novel immunotherapies targeting CHB and its progression to hepatocellular carcinoma.

## Materials and methods

### Cell lines and monoclonal antibodies

Expi293F™ GnTI- and ExpiCHO-S™ cell lines were obtained from Thermo Fisher Scientific Inc. and used for protein expression. The anti-CD33 monoclonal antibody (10C8) was generated from a phage-displayed synthetic antibody library, as previously described [29].

### Cloning, expression, and purification of human CD33-ECD and Fab-10C8

The CD33 extracellular domain (CD33-ECD, residues 1–232) was expressed as a stand-alone protein to enable co-purification with the Fab-10C8. The CD33-ECD.Fc fusion protein was generated as previously described [29]. For Fab co-purification, CD33-ECD was expressed via transient transfection of Expi293F™ GnTI-cells cultured in suspension in Expi293 medium (Thermo Fisher Scientific), following the manufacturer’s instructions. Culture supernatants were harvested 5 days post-transfection and filtered prior to loading onto an affinity column with Fab-His₆.

The Fab-10C8 was produced by introducing a C-terminal His₆ tag on the heavy chain. Fab-His₆ was expressed in ExpiCHO-S™ cells cultured in ExpiCHO medium (Thermo Fisher Scientific) and harvested 8 days post-transfection. The Fab-containing supernatant was filtered and loaded onto a 5 mL pre-washed HiTrap Excel column (Cytiva) at 4 °C, followed by loading of the filtered CD33-ECD supernatant onto the same column to facilitate co-purification. Protein elution was performed on an ÄKTA Go system using a four-step imidazole gradient (20, 50, 100, and 200 mM) in buffer containing 100 mM Tris–HCl (pH 8.5) and 500 mM NaCl. The CD33-ECD/Fab-His₆ complex was eluted in buffer containing 100 mM imidazole, 100 mM Tris–HCl (pH 8.5), and 500 mM NaCl. The eluted complex was concentrated to 2 mL using a 30 kDa MWCO centrifugal device (Millipore) and subjected to size-exclusion chromatography using a Superdex 200 Increase 10/300 GL column (Cytiva) pre-equilibrated with PBS (pH7.4) at a flow rate of 0.5 mL/min. Fractions containing the CD33-ECD/Fab-10C8 complex were pooled and concentrated to 5 mg/mL for further analysis.

### Sedimentation velocity analytical ultracentrifugation (AUC-SV)

Analytical ultracentrifugation (AUC) experiments were performed using a Beckman-Coulter XL-A analytical ultracentrifuge equipped with a Ti An60 rotor. Sedimentation velocity (SV) analysis was conducted to characterize the oligomeric states and molecular weights of CD33-ECD, Fab-10C8, and the CD33-ECD/Fab-10C8 complex. All protein samples were prepared in phosphate-buffered saline (PBS), with individual components tested at a concentration of 0.5 mg/mL. The density and viscosity of the buffer were determined using the SEDNTERP program. Centrifugation was carried out at 20 °C, with CD33-ECD sedimented at 60,000 rpm, while Fab-10C8 and the CD33-ECD/Fab-10C8 complex were sedimented at 50,000 rpm. Sedimentation profiles were recorded in real-time by monitoring the absorbance at 280 nm. The resulting data were analyzed with the SEDFIT program to fit a continuous sedimentation coefficient distribution model, *c*(*s*), by numerically solving the Lamm equation.

### Surface plasmon resonance (SPR) binding analysis

Binding kinetics of the interaction between the human CD33-Fc fusion protein and anti-CD33 mAb (clone 10C8) were characterized by surface plasmon resonance (SPR) using a Biacore T200 system (Cytiva). The anti-CD33 mAb was covalently immobilized onto a CM5 sensor chip (Cytiva) via standard amine coupling. Kinetic measurements were performed by injecting hCD33-Fc at concentrations of 1, 5, 10, 50, and 100 nM in HBS-EP^+^ running buffer (Cytiva) over the functionalized sensor surface. A duplicate injection of the 10 nM concentration was included to ensure surface stability and reproducibility. All experiments were conducted at 25 °C. The resulting sensorgrams were processed using the Biacore Evaluation Software (Cytiva). Kinetic parameters, including the association rate constant (ka), dissociation rate constant (kd), and equilibrium dissociation constant (KD), were determined by globally fitting the data to a 1:1 Langmuir binding model.

### Crystallization and X-ray data collection

The CD33-ECD/Fab-10C8 complex was concentrated to 5 mg/mL and subjected to crystallization screening using the hanging-drop vapor diffusion method. Crystallization drops were set up with a Mosquito Crystal robot (SPT Labtech) by mixing 0.1 μL of protein solution with 0.1 μL of reservoir solution. Optimized crystals were obtained in a condition containing 15% PEG 4000, 0.2 M MgCl_2_, 100 mM Tris–HCl (pH 8.5), and 20 mM NaBr. Crystals were cryoprotected by brief soaking in mother liquor supplemented with 20% PEG 4000 and flash-cooled in liquid nitrogen. X-ray diffraction data were collected at the TPS05A beamline of the National Synchrotron Radiation Research Center (NSRRC, Taiwan) at a wavelength of 0.99984 Å. The crystals diffracted to 3.2 Å resolution and belonged to space group P1, with unit cell dimensions *a* = 71.4 Å, *b* = 71.2 Å, *c* = 86.8 Å, *α* = 95.9°, *β* = 94.2°, *γ* = 111.2° (Supplementary Table S1). The asymmetric unit contained two CD33-ECD/Fab-10C8 complexes.

### Structure prediction, molecular replacement, and model refinement

X-ray diffraction data for the CD33-ECD/Fab-10C8 complex were processed using DENZO and SCALEPACK in the HKL2000 package [30]. The crystals exhibited anisotropic diffraction, characterized by an ellipsoidal diffraction pattern. To determine the optimal resolution limit, we systematically evaluated various cutoffs (3.8–2.8 Å) by monitoring refinement stability and map quality. The final resolution was set at 3.2 Å based on the *CC*_1/2_ values (0.97 in the highest shell), *I/σ* (6.4 in the highest shell), and the convergence of *R*_*free*_ statistics. Despite the anisotropic nature of the diffraction, this cutoff yielded a dataset with acceptable completeness (71.6% in the highest resolution shell) and well-defined electron density maps. The diffraction data were initially analyzed using *phenix.xtriage* and *CCP4 Zanuda* [31–33], both of which suggested C2 space group due to apparent pseudo-symmetry. However, integration and scaling attempts in C2 resulted in the systematic rejection of a substantial fraction of reflections, particularly in the intermediate resolution range (3.2–5.0 Å). In contrast, processing the data in P1 allowed for the successful integration of the full diffraction range, yielding a more complete and internally consistent dataset (Supplementary Table S1). Furthermore, refinement in P1 produced well defined electron density maps and improved *R-factors* without any indications of overlooked crystallographic symmetry. Therefore, P1 was selected as the final space group to ensure the highest data quality and model reliability. Initial molecular replacement (MR) attempts using existing CD33-ECD monomer or dimer structures and homologous Fab templates as search models failed to yield a correct solution. To generate a suitable MR model, AlphaFold2 predictions were performed using the ColabFold pipeline by inputting the sequences of CD33-ECD (residues 18–232), Fab heavy chain, and Fab light chain. The resulting predicted complex model, with a Z-score of 0.9, successfully produced correct phases in *Phaser* (PHENIX suite) [31, 32]. Manual model building was conducted in *Coot* [34]. Model refinement was performed using *PHENIX* with a protocol including coordinate refinement, individual atomic B-factor refinement, and occupancy refinement. No additional custom restraints, such as non-crystallographic symmetry (NCS) or secondary structure restraints, were manually applied. *N*-linked *N*-acetylglucosamine residues were modeled and linked to Asn side chains using the carbohydrate module in *Coot* [35]. The final model was refined to 3.2 Å resolution, with *R* and *R*_*free*_ values of 20.5% and 25.2%, respectively (Supplementary Table S1). The electron density was well resolved for both Fab chains and the CD33 V-set and C2-set domains. PyMOL was used for structural analysis and figure preparation. Buried surface area values were calculated using the PDBePISA server (EMBL-EBI) [36]. Coordinates and structure factors have been deposited in the Protein Data Bank under accession code 9VL2.

### Negative-stain electron microscopy

To assess the assembly of the CD33-ECD/Fab-10C8 complex in solution, negative-stain electron microscopy was performed. The complex was obtained from fraction 16 of the size-exclusion chromatography (Supplementary Fig. S1) and adjusted to a final concentration of 0.06 mg/mL. A droplet of the sample was placed on a clean parafilm surface and then picked up onto a carbon-coated grid before being negatively stained with 1% uranyl acetate. After the grid had been air-dried for 1 day, images were captured using a Talos 120 C electron microscope (Thermo Fisher Scientific) operated at 120 kV, equipped with a single-tilt holder and a Thermo Ceta 16 M camera. Image frames were randomly selected from different grids to evaluate the overall architecture and stoichiometry of the complex.

## Result

### Formation and characterization of the CD33-ECD/Fab-10C8 complex

Previous studies have shown that CD33 binds HBV surface antigen (HBsAg) through α2,6-linked sialoglycans, leading to ITIM phosphorylation and SHP-1/2 recruitment, which suppresses myeloid cell activation [29]. In our previous work, we identified the monoclonal antibody 10C8 as a potent antagonist of CD33-mediated immunosuppression [29]. 10C8 was shown to restore cytokine responses in PBMCs from patients with chronic HBV infection by blocking the CD33-HBsAg interaction. Despite its promising immunomodulatory activity, the structural basis for 10C8 recognition and inhibition of CD33 remains unknown.

**Fig. 1:**
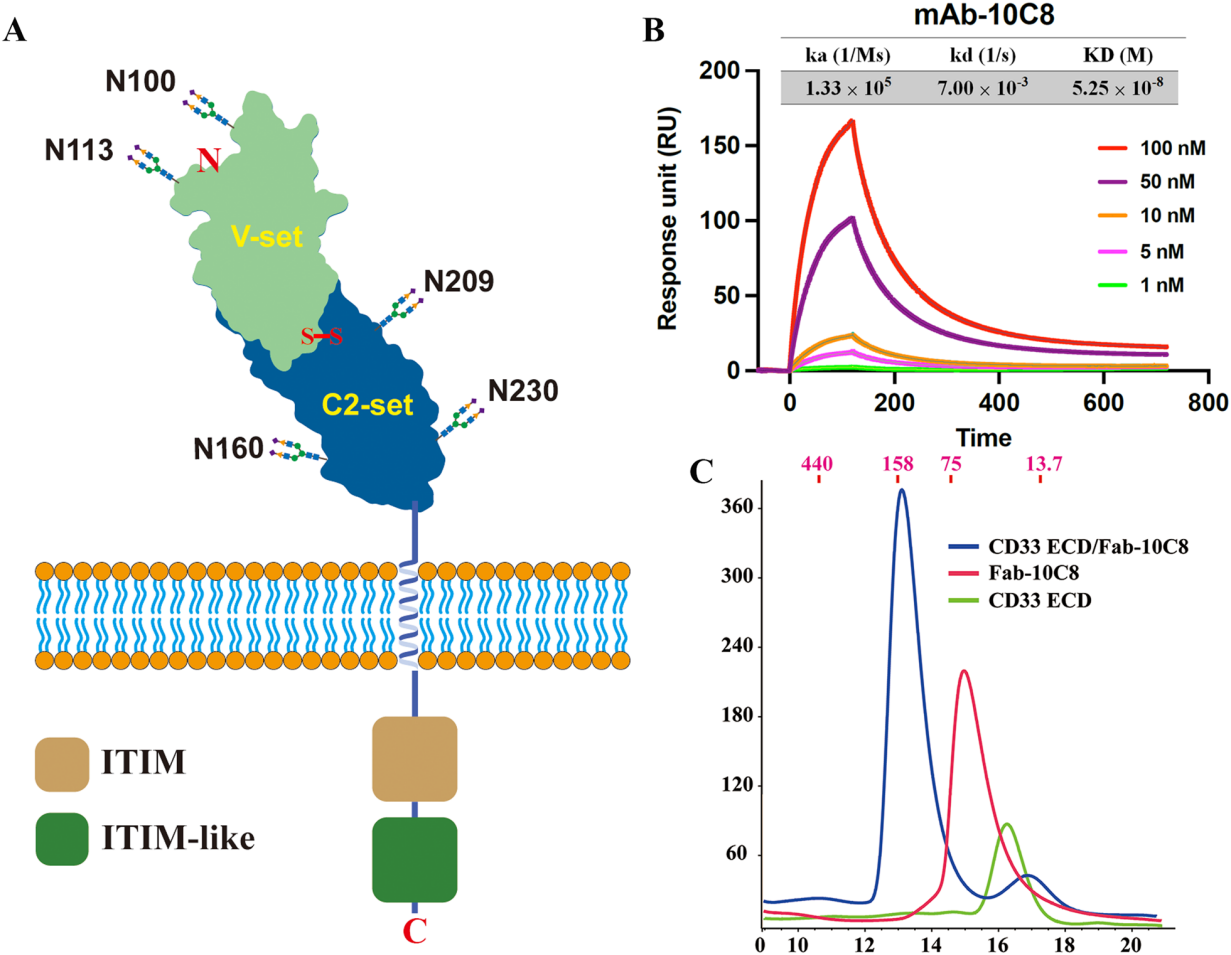
Structural overview of CD33 and formation of a stable CD33–10C8 complex. **A** Domain organization of human CD33 (SIGLEC-3), showing the extracellular IgV and IgC2 domains, single transmembrane (TM) helix, and cytoplasmic tail containing ITIM and ITIM-like motifs. The conserved *N*-linked glycosylation sites are indicated, as reported in previous studies [29, 41]. **B** Surface plasmon resonance (SPR) analysis of 10C8 binding to the human CD33 extracellular domain. Kinetic sensorgrams show the binding response at various concentrations (1, 5, 10, 50, and 100 nM). The derived kinetic parameters, including the association rate constant (ka), dissociation rate constant (kd), and equilibrium dissociation constant (KD), are summarized in the inset table. **C** Size-exclusion chromatography (SEC) analysis demonstrating the formation of a stable complex between CD33-ECD (residues 18–232) and the anti-CD33 monoclonal antibody Fab-10C8. Proteins were analyzed using a Superdex 200 Increase 10/300 GL column (Cytiva) equilibrated in PBS buffer. The elution traces of CD33-ECD (green), Fab-10C8 (red), and the 1:1 molar mixture (blue) are shown. The complex elutes at a significantly earlier volume compared to either individual component, indicative of a stable, higher-order assembly. Molecular weight standards (440, 158, 75, and 13.7 kDa) are indicated at the top in magenta

As illustrated in Fig. 1A, CD33 adopts a two-domain Ig-like architecture consisting of an N-terminal V-set domain (residues 18–140) and a membrane-proximal C2-set domain (residues 141–232), connected by a short linker region (residues 140–142). These extracellular domains are followed by a single transmembrane helix and cytoplasmic ITIM and ITIM-like motifs that mediate inhibitory signaling [37]. Notably, sequence analysis identifies five potential *N*-linked glycosylation sites located at Asn100 and Asn113 within the V-set domain, and at Asn160, Asn209, and Asn230 within the C2-set domain [29]. Because of the heterogeneous occupancy and varying mass of these glycans, molecular weight estimates for the CD33-containing complexes in solution are fundamentally approximate.

The binding affinity between 10C8 and CD33 was first quantitatively characterized using surface plasmon resonance (SPR) analysis to provide a biophysical benchmark for the interaction (Fig. 1B). The 10C8 monoclonal antibody exhibits robust binding to the human CD33 extracellular domain with an equilibrium dissociation constant (KD) of 5.25 × 10^−8^ M (52.5 nM). The kinetic analysis yielded an association rate constant (ka) of 1.33 × 10^5^ M^−1^ s^−1^ and a dissociation rate constant (kd) of 7.0 × 10^−3^ s^−1^, confirming a stable interaction between the antibody and its target.

For subsequent structural studies, the mature human CD33 extracellular domain (ECD; residues 18–232) was expressed in Expi293F™ cells and purified to assemble the complex with the Fab-10C8. To obtain a homogeneous complex suitable for crystallization, the CD33-ECD/Fab-10C8 mixture was subjected to gel filtration chromatography. As shown in Fig. 1C, both CD33-ECD (~24 kDa) and Fab-10C8 (~49 kDa) eluted as single, symmetrical peaks when analyzed individually by size-exclusion chromatography, suggesting their monomeric states in solution. In contrast, the mixture of CD33-ECD and Fab-10C8 showed a distinct earlier-eluting peak relative to either component alone, indicative of complex formation. To precisely estimate the size of the resulting complex, we performed SEC with a molecular weight standard calibration (Supplementary Fig. S1). Based on this calibration, the elution peak of the CD33-ECD/Fab-10C8 complex corresponds to an apparent molecular weight of approximately 132 kDa. While this value is slightly lower than the theoretical mass of a 2:2 stoichiometry (146 kDa), it clearly deviates from the ~73 kDa expected for a simple 1:1 complex. The minor discrepancy in SEC-derived mass is likely due to the non-globular, elongated geometry of the assembly and the influence of *N*-glycans on CD33-ECD, both of which affect the hydrodynamic radius.

The stoichiometry of the resulting assembly was further validated by sedimentation velocity analytical ultracentrifugation (AUC-SV), which serves as an additional approach for estimating the molecular weight, further supporting the formation of a 2:2 complex in solution. CD33-ECD and Fab-10C8 exhibited sedimentation coefficients of approximately 3.7 S and 4.4 S, corresponding to molecular weights of 26.6 kDa and 48.7 kDa, respectively. Upon complex formation, a shifted peak at approximately 5.7 S was observed, yielding a consistent molecular weight of 157 kDa (Supplementary Fig. S2), further supporting that the CD33-ECD/Fab-10C8 complex exists as a 2:2 stoichiometry in solution. This estimation of molecular mass aligns with a model where two CD33-ECD molecules are each bound by a separate Fab fragment, forming a higher-order assembly that accounts for the earlier elution observed in SEC. These results establish the stoichiometry of the complex, which serves as the biophysical basis for our subsequent structural analysis. Interestingly, previous studies have reported dimeric behavior of CD33 in various contexts. Immunoprecipitation-based analyses suggested that CD33 forms dimers on the cell surface [37]. In addition, the crystal structure of CD33 (PDB: 5IHB) revealed a dimeric arrangement in the asymmetric unit, and an independent size-exclusion chromatography study has also demonstrated the propensity of the isolated CD33 IgC1 domain to form homodimers in solution [38]. In contrast, our purified human CD33-ECD behaved as a monomer in solution (Fig. 1C). Taken together, these observations raise the possibility that binding of the Fab-10C8 modulates the dimerization propensity of CD33-ECD in solution, either by stabilizing a transient 2:2 arrangement or by inducing a distinct higher-order complex that is not observed in the unbound state.

### Overall structure of CD33-ECD/Fab-10C8 complex

**Fig. 2:**
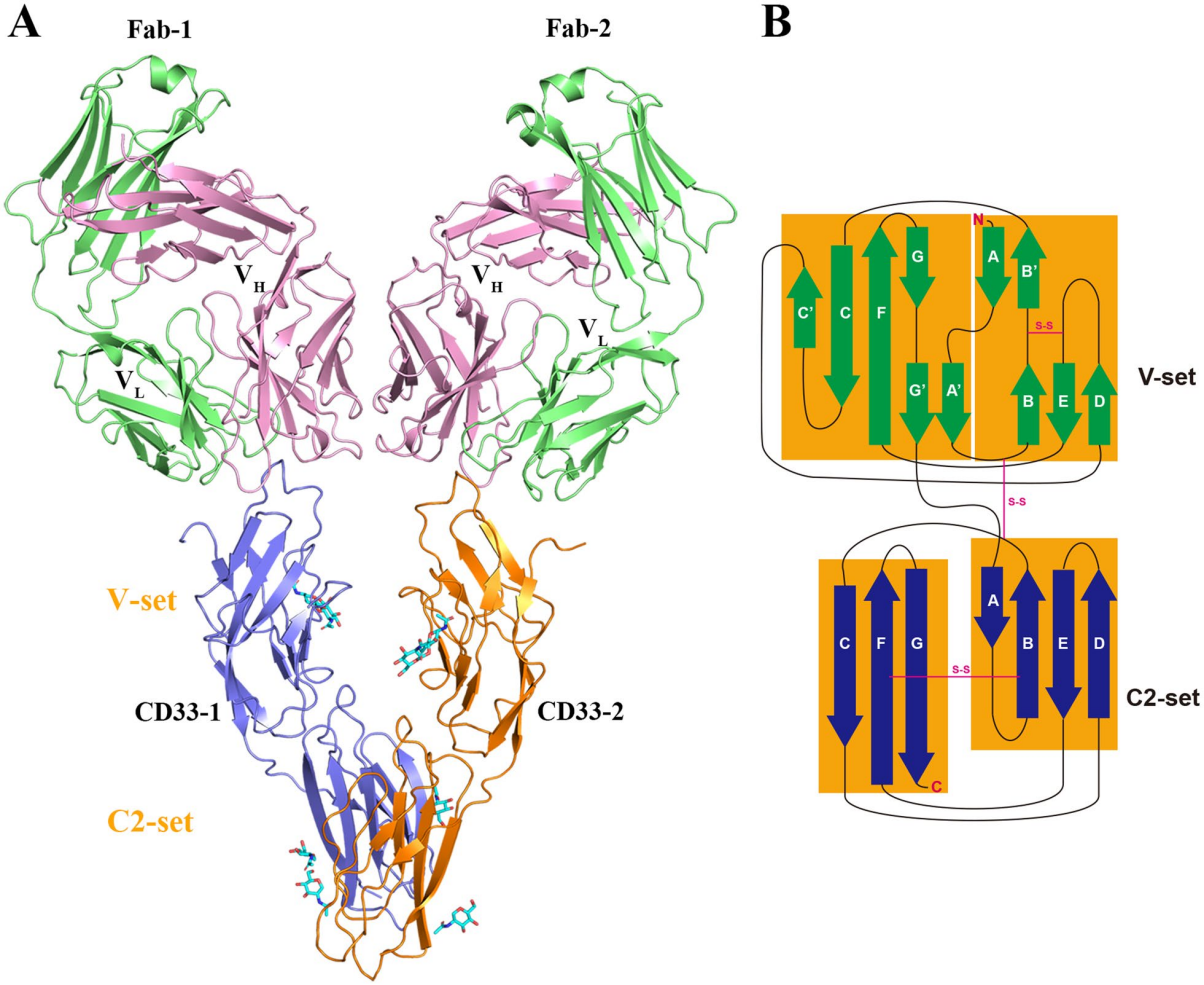
Crystal structure of the CD33-ECD/Fab-10C8 complex. **A** Overall structure of the CD33 extracellular domain (residues 18–232) in complex with two Fab-10C8. CD33 monomers are colored in blue (CD33-1) and orange (CD33-2), respectively, with the V-set and C2-set domains labeled. The Fab heavy chains (HC) and light chains (LC) are colored in pink and green, respectively. *N*-linked glycans are displayed as cyan sticks. **B** Topology diagram of the CD33 extracellular domain (ECD), illustrating the organization of the N-terminal V-set and membrane-proximal C2-set Ig-like domains. Disulfide bonds are indicated, reflecting conserved intramolecular linkages that stabilize the immunoglobulin fold

While the functional antagonism of 10C8 has been established, the precise structural mechanism by which it exerts this effect remains unclear. To address this, we determined the crystal structure of the CD33-ECD/Fab-10C8 complex obtained from gel filtration-purified samples and diffracted to 3.2 Å resolution. The structure was solved by molecular replacement and revealed an asymmetric unit composed of two CD33-ECD molecules and two Fab-10C8 antibodies (Fig. 2A). Clear electron density was observed for both CD33 and Fab components, confirming high-quality maps suitable for model building (Supplementary Fig. S3). The overall conformation of the two CD33-ECD/Fab-10C8 pairs is highly similar, with an average root-mean-square deviation (RMSD) of 0.88 Å. The major differences are confined to the C2 domain, suggesting flexibility in the interdomain connection (Supplementary Fig. S4). At the antigen-binding interface, the two Fab molecules exhibit highly similar complementarity-determining region (CDR) loop conformations.

As shown in Fig. 2A, the quaternary structure arrangement adopts a pseudo twofold symmetric architecture. Each CD33-ECD molecule is bound independently by a Fab-10C8 antibody on its V-set domain. The two Fabs extend outward in opposite directions, generating a V-shaped configuration. The variable domains in the Fab heavy chain (V_H_) are positioned in close proximity and form direct inter-Fab interactions, which likely contribute to the stability of the 2:2 complex observed in the crystal (Fig. 2A).

Each CD33-ECD monomer adopts a two-domain Ig-like fold consisting of an N-terminal V-set domain (residues 18–140) and a membrane-proximal C2-set domain (residues 141–232), connected by a short linker (residues 140–142). The V-set domain consists of two antiparallel β-sheets comprising β-strands ABB′ED and A′GG′FCC′, stabilized by an intradomain disulfide bond (Cys41–Cys101). The C2-set domain forms two β-sheets composed of strands ABED and CFG, and is stabilized by an internal Cys163–Cys212 disulfide bond (Fig. 2B).

In addition, CD33-ECD has five *N*-linked glycans located at Asn100 and Asn113 in the V-set domain, and Asn160, Asn209, and Asn230 in the C2-set domain. Electron density for glycans was observed at Asn100, Asn160 and Asn209 in both CD33-ECD molecules (Supplementary Fig. S5), whereas the remaining sites appear too flexible to be resolved.

In this complex structure, two CD33-ECD molecules interact through an asymmetric interface formed by the βC strands of each C2-set domain. The hydrophobic residues of the βC strand from CD33-1 participate in the hydrophobic core composed of the βC, βD, and βE strands of CD33-2, with reciprocal contributions from both protomers. This interface buries approximately 763.7 Å^2^ of solvent-accessible surface area and is stabilized by a network of backbone hydrogen bonds, hydrophobic side-chain contacts, and polar interactions (Supplementary Table S2). Notably, the βC strands of each monomer are arranged in an antiparallel fashion reminiscent of a zipper (Supplementary Fig. S6). Comparison between the two monomers in our CD33-ECD structure indicates that no significant rearrangements occur between two monomers.

**Fig. 3:**
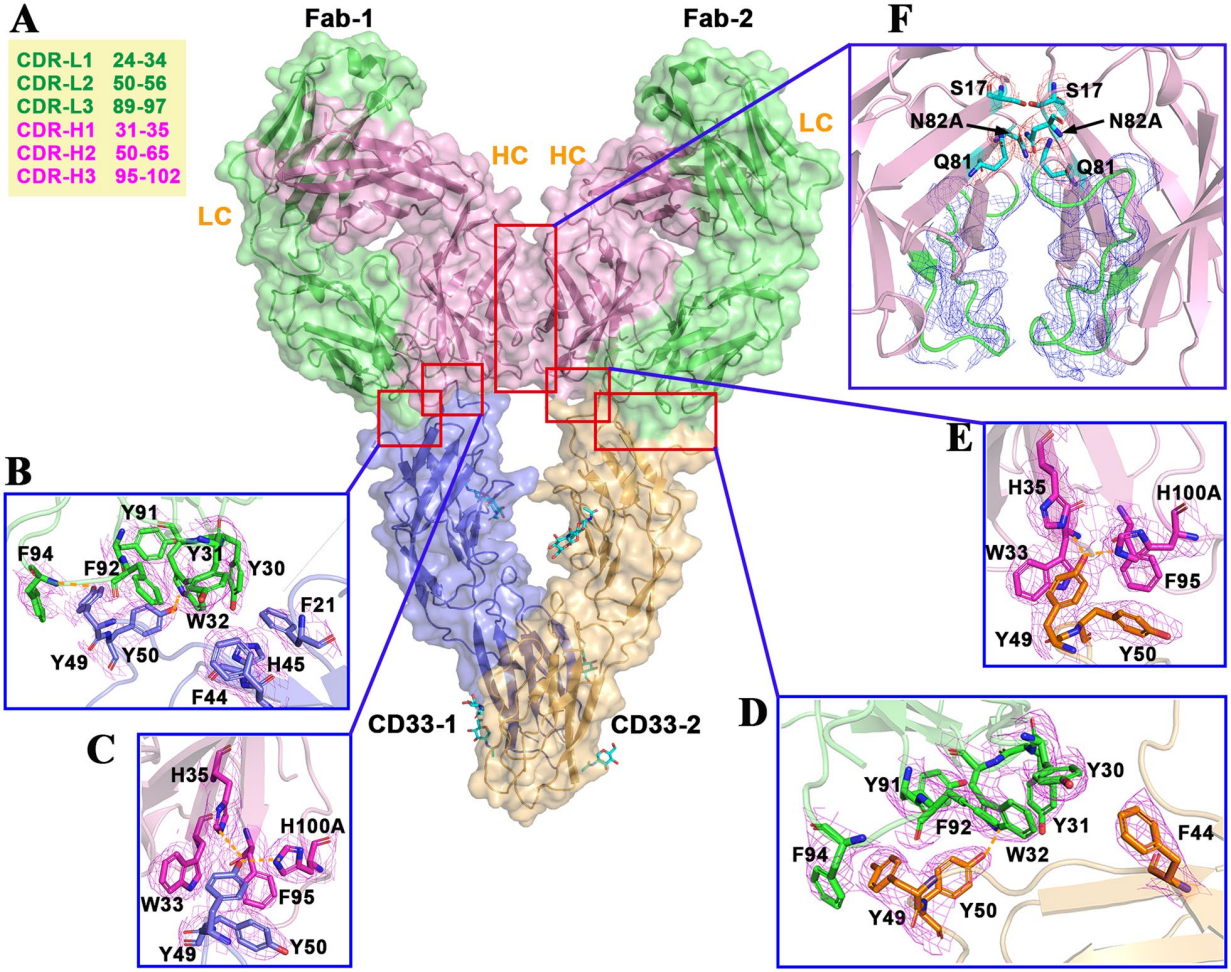
Structural basis of epitope recognition by anti-CD33 antibody 10C8. The extracellular domain of CD33 (residues 18–232) is shown in complex with two Fab-10C8. CD33 monomers (CD33-1 and CD33-2) are colored in blue and orange, while Fab light (LC) and heavy (HC) chains are shown in green and pink, respectively. **A** Overview of the complex with the CDR loops highlighted in color (CDR-L1-3, CDR-H1-3). **B**,**D** Close-up views of the interaction interfaces between the CD33 V-set domain and the heavy-chain CDRs of Fab-1 and Fab-2. **C**,**E** Corresponding views of the interactions with the light-chain CDRs. **F** Additional polar contacts involving VH–VH inter-Fab interface are highlighted. The 2*Fo*-*Fc* electron density map contoured at 0.8 σ

Each Fab-10C8 antibody consists of a complete light chain (LC; residues 1–217) and a partial heavy chain (HC; residues 1–223), forming a canonical four-domain immunoglobulin (Ig) fold (Fig. 2A). Each domain contains a conserved intradomain disulfide bond that stabilizes the β-barrel architecture. Although most regions were well resolved in the electron density, residues 134–140 in heavy chain domain appeared disordered. The complementarity-determining regions (CDRs) are hypervariable loops that form the antigen-binding site of antibodies. The identification of these CDR loops and all associated residue numbering follow the standard Kabat convention (Fig. 3A). These loops cluster at the tip of the variable domains to define the antigen-binding surface.

### CD33-ECD forms a stable complex with Fab-10C8 antibody through a V-set epitope

The two CD33-ECD/Fab-10C8 pairs adopt nearly identical binding modes, consistent with the pseudo-symmetric arrangement of the complex (Fig. 3). In this structure, each Fab molecule recognizes a defined epitope on the CD33 V-set domain primarily through CDR1 and CDR3 of both chains, while CDR2 is excluded from direct involvement (Fig. 3). The CD33-ECD binding surface is primarily composed of βC and βD and their adjacent loops in the V-set domain, providing the antigenic platform recognized by these CDR loops. Within the 2:2 stoichiometric assembly, two independent Fab-10C8 molecules bind to the CD33-ECD dimer, generating two distinct binding interfaces that engage both CDR-L and CDR-H regions (Fig. 3). Interface analysis using the PDBePISA server reveals a similar core binding mode across both interfaces. Although Fab-2 achieves a slightly larger overall buried surface area (612.6 Å^2^ vs. 542.6 Å^2^), CD33-1 engages additional peripheral contacts outside the core interface, reflecting subtle but complementary differences in the geometry of each binding interaction. In both cases, the light and heavy chains contribute consistently to antigen recognition, with light chain residues providing the major structural contacts. On the antibody side, both Fab-1 and Fab-2 engage CD33-ECD through a similar network of interactions (Fig. 3B–E). The light chain contributes Tyr30, Tyr31, and Trp32 from CDR-L1, together with Tyr91, Phe92, and Phe94 from CDR-L3 (Fig. 3B, D). On the heavy chain, Trp33, Phe95, and His100A from CDR-H1/3 contact CD33 Tyr49 and Tyr50, while the heavy chain framework residue His35 establishes a hydrogen bond with Tyr49 of CD33 (Fig. 3C, E). This combination of CDR- and framework-derived residues yields a tightly packed and complementary interface. The prominent enrichment of bulky aromatic side chains (especially Tyrosine) at the binding interface favorably contributes to the interaction; consistent with previous structural studies [39], these bulky residues provide extensive buried surface area and versatile non-covalent interactions (including aromatic stacking and hydrogen bonds) that uniquely anchor the Fab onto the CD33 β-sheet platform. Notably, the interface is dominated by aromatic stacking and hydrophobic contacts, particularly between CDR-L1 and CDR-L3 (Tyr30, Trp32, Phe94) and the CD33 B′-C loop (Tyr49, Tyr50), with a few hydrogen bonds (such as Phe94-Tyr49) providing additional stabilization (Fig. 3B–E).

In addition to antigen recognition, the two Fab molecules interact directly through their heavy chain variable domains (Fig. 3F). This VH–VH interface involves residues Ser17, Gln81, and Asn82A, which are conserved across human VH germline sequences (Supplementary Fig. S7). This inter-Fab interaction might further stabilize the 2:2 assembly, complementing the CD33-ECD/Fab-10C8 contacts. The *N*-linked glycans observed at Asn100, Asn160, and Asn209 are positioned on solvent-exposed surfaces distal from both the antibody-binding site and the dimerization interface, without engaging in any direct contacts with either the Fab or the C2-set domains (Fig. 2A).

To further validate whether this 2:2 assembly is preserved in solution, we performed negative-stain electron microscopy experiment on the purified CD33-ECD/Fab-10C8 complex. The representative EM images revealed readily identifiable particles adopting a compact, V-shaped architecture (Supplementary Fig. S8). Notably, the dimensions of these individual particles (approximately 130 Å in length and 100 Å in width) closely match the overall size and surface representation of the 2:2 heterotetrameric complex observed in our crystal structure (Supplementary Fig. S8). Taken together, these findings demonstrate that Fab-10C8 binds CD33 through a nearly identical V-set epitope with symmetric geometry and forms inter-Fab interactions that reinforce the dimeric CD33-ECD configuration, suggesting a potential mechanism by which Fab-10C8 may stabilize CD33 dimers in solution and thereby influence downstream signaling.

### Fab binding induces a conformational shift in the CD33 dimer

To explore the structural basis of Fab-10C8-mediated antagonism, we compared our CD33-ECD/Fab-10C8 complex with the previously reported apo structure of CD33 (PDB: 5IHB). This comparison enabled assessment of potential Fab-induced conformational changes, which may contribute to the antagonistic activity of 10C8. Specifically, we examined alterations in domain orientation, loop flexibility, and dimerization geometry.

**Fig. 4:**
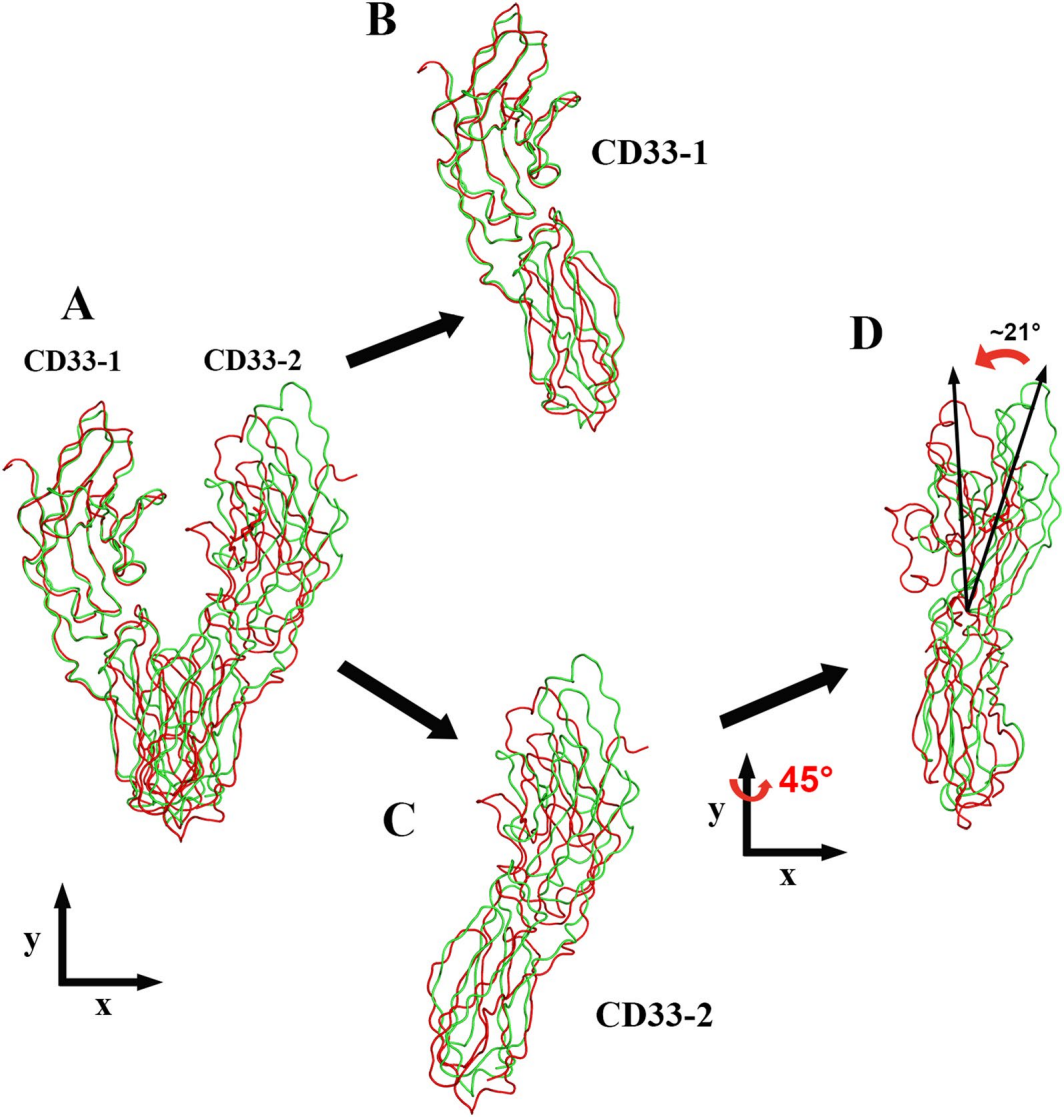
Structural comparison between Fab-bound and *apo* CD33 reveals a potential Fab-induced compaction of the dimeric ectodomain. **A** Superposition of Fab-bound CD33-ECD (red) and apo CD33 (green; PDB: 5IHB), aligned using the V-set and C2-set domains of CD33-1. **B** The V-set domains overlay closely, whereas the C2-set domain of CD33-1 displays a measurable displacement (RMSD = 2.20 Å). **C** A 90° rotation along the *y*-axis reveals that CD33-2 undergoes a more pronounced inward shift, resulting in narrowing of the interdomain angle. **D** A further 45° tilt highlights both the local movement of CD33-2 relative to CD33-1 and an overall ~21° reduction of the dimer angle. *Apo* and Fab-bound structures are shown as green and red ribbons, respectively

Structural alignment was performed using the V-set and C2-set domains of CD33-1 as a reference points (Fig. 4A). While the V-set domains of Fab-bound and unbound CD33 superimpose closely, the C2-set domain shows a measurable displacement, with an RMSD of 2.2 Å (Fig. 4B). In CD33-2, this displacement is observed as a more pronounced inward shift, leading to ectodomain compaction and narrowing of the interdomain angle (Fig. 4C). These conformational differences become clearer after a 45° rotation along the *y*-axis. This observation highlights the local inward movement of CD33-2 relative to CD33-1, in contrast to the ~21° narrowing of the dimer angle (Fig. 4D). Collectively, these analyses indicate that Fab binding stabilizes a more compact ectodomain conformation compared to the relatively open dimer observed in the apo structure.

This conformational rearrangement appears to be driven by substantial inter-Fab interactions involving the variable domains. Remarkably, the buried surface area at the Fab-Fab interface (404.6 Å^2^) is substantial and comparable in scale to antigen-contact regions, underscoring the critical role of these inter-Fab contacts in stabilizing the complex. Three key hydrogen bonds stabilize this contact: (1) Asn82A (Fab-1-HC ND2) to Gln81 (Fab-2-HC OE1), (2) the reciprocal Asn82A-Gln81 interaction in trans, and (3) Ser17 (Fab-2-HC) to Ser17 (Fab-1-HC) (Fig. 3F). Additional stabilization arises from hydrophobic and van der Waals interactions spanning residues 56–60 and 68–78 of the two heavy chains. Together, these forces draw the Fab molecules toward one another, pulling the V-set domains of CD33 inward and producing a reduced V-shaped opening (Fig. 4C, D). Our previous study demonstrated that α2,6-linked sialoglycans on HBsAg induce CD33-mediated ITIM phosphorylation and SHP-1/2 recruitment, leading to immune suppression in CHB patient-derived PBMCs [29]. Notably, 10C8 reversed this inhibition by restoring cytokine production [29]. The potential Fab-induced inward shift of the CD33 ectodomains, along with reduced interdomain flexibility, may allosterically influence the accessibility or alignment of the intracellular ITIM/ITIM-like motifs. This conformational restriction could potentially reduce SHP-1/2 engagement, suggesting a structural explanation for the antibody’s antagonistic effect.

Together, these results support a model in which 10C8 stabilizes a structurally compact, less permissive CD33 conformation, providing a plausible structural basis for modulation of inhibitory signaling through ectodomain-driven allosteric effects.

## Discussion

Prior studies first identified 10C8 as an antagonistic anti-CD33 monoclonal antibody using phage-displayed combinatorial libraries [29]. In a mast cell degranulation model, TriNitroPhenol-conjugated liposomes (TNP-LPs) triggered robust degranulation, which was significantly suppressed by co-administration of synthetic CD33 ligand-containing liposomes. Notably, this suppression was effectively reversed by 10C8, whereas an isotype control antibody showed no effect [29]. These findings provided early functional evidence that 10C8 could antagonize CD33-mediated inhibitory signaling induced by sialoglycan ligands.

Our structure reveals a highly ordered 2:2 CD33-Fab complex, stabilized not only by receptor-receptor interactions but also by inter-Fab contacts. This conformation is geometrically constrained, compact, and unlikely to support further lateral assembly or clustering. We therefore propose that 10C8 stabilizes CD33 in a non-productive dimeric conformation, thereby preventing glycan-mediated receptor aggregation and subsequent inhibitory signaling. Such a mechanism offers a structural explanation for the antibody’s ability to reverse CD33-dependent immunosuppression in chronic hepatitis B. This clustering-dependent signaling paradigm is well established across multiple Siglec family members, including CD22, Siglec-7, and CD33, where ligand multivalency facilitates ITIM phosphorylation via spatial concentration of signaling motifs [15].

In addition, compared to the previously reported apo-CD33 structure (PDB: 5IHB), our Fab-bound complex exhibits a more compact and symmetric dimeric architecture (Fig. 4). In the apo state, CD33 molecules adopt a more relaxed orientation with considerable inter-domain flexibility, whereas 10C8 engagement induces a pronounced inward movement of one protomer and stabilizes inter-Fab contacts [38]. We found that CDR-H2, which does not directly participate in the CD33-ECD/Fab-10C8 interaction, is located near the inter-Fab contacts (Fig. 3F). Although the two CDR-H2 regions do not interact with each other in this complex structure, their proximity raises the possibility that CDR-H2 positioning could influence the strength of inter-Fab contacts when different antibodies are involved. Consequently, this may modulate the intensity of the immune response and the magnitude of the downstream cellular signaling events. Such antibody-induced compaction likely constrains ectodomain geometry, reducing the conformational plasticity required for ligand-induced clustering and downstream signaling. These findings support the model in which antagonistic antibodies restrict CD33 into a signaling-refractory conformation.

**Fig. 5:**
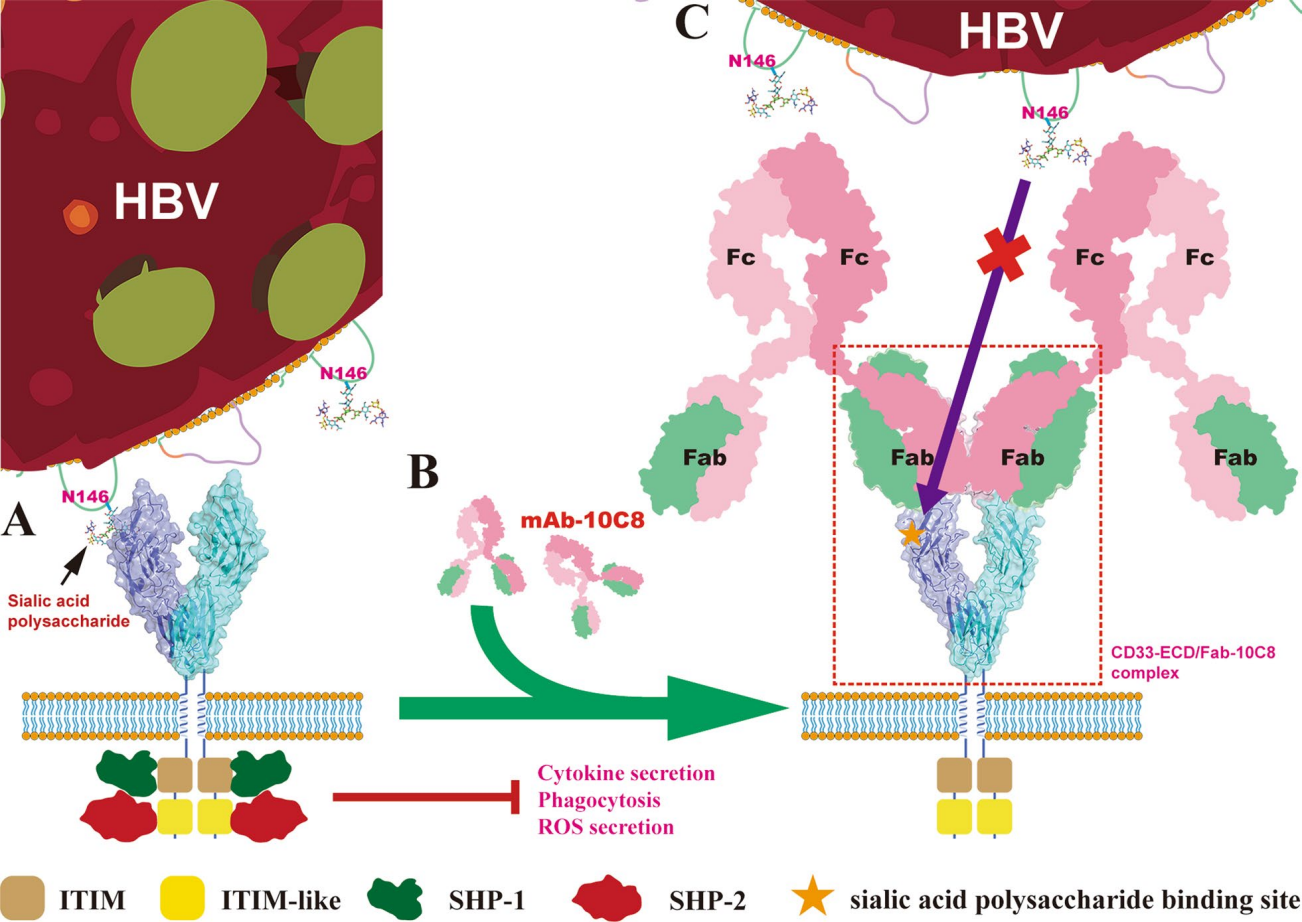
Proposed mechanism of 10C8-mediated immunomodulation via CD33 blockade. **A** Canonical inhibitory signaling: In the absence of antibody, CD33 engages the α2,6-linked sialoglycan at Asn146 of HBsAg through its V-set domain. This interaction triggers ITIM phosphorylation, leading to SHP-1/2 recruitment and subsequent suppression of effector functions, including cytokine secretion, phagocytosis, and ROS production. **B** Steric occlusion: mAb-10C8 binds to the CD33 V-set domain with high affinity. Although the 10C8 epitope does not directly overlap with the canonical sialic acid-binding pocket, the antibody’s lateral orientation effectively blocks HBsAg access through steric hindrance. **C** Conformational locking and signaling blockade: The formation of a stable 2:2 stoichiometry complex induces a more compact dimeric arrangement of CD33 (marked by a ~21° reduction in the dimer angle). This rigid, antibody-enforced conformation restricts the conformational plasticity required for receptor clustering and efficient SHP-1/2 recruitment, thereby neutralizing HBV-induced immunosuppression and restoring myeloid cell activation. The red dashed box denotes the region corresponding to the experimentally determined crystal structure of the CD33-ECD/Fab-10C8 complex reported in this study

Based on these data and the CD33-ECD/Fab-10C8 complex structure, we propose a model for how 10C8 antibody influences HBV binding to CD33 (Fig. 5). As shown in Fig. 5A, HBV virions presenting α2,6-linked sialoglycans (notably at Asn146 on HBsAg) engage CD33, facilitating receptor clustering that promotes ITIM/ITIM-like motif phosphorylation and SHP-1/2 recruitment, thereby suppressing immune effector functions. In Fig. 5B, the introduction of 10C8 competitively displaces HBV through high-affinity binding to the B′-C and F-G loops of the CD33 V-set domain. This binding induces a compact dimeric conformation that is both sterically and conformationally restricted, thereby precluding further clustering or engagement with multivalent glycan ligands. Functionally, this structural intervention is consistent with interruption of inhibitory signal transduction and restoration of immune cell activity.

The antagonistic mechanism operates through two synergistic modes (Fig. 5C). First, although the 10C8 epitope does not directly overlap the canonical sialic acid-binding groove centered at Arg119, the Fab variable domains are positioned adjacent to the F-G region and the C-strand, where key glycan-interacting residues (Tyr127, Arg119, His45) reside. The lateral orientation and bulk of the Fab framework and CDR loops impose spatial occlusion over the B′-C and F-G loop regions critical for glycan recognition (Fig. 2). While small sialoglycans might theoretically access the binding pocket, the ~50 kDa Fab fragment creates a substantial barrier that physically precludes large HBsAg-decorated virions from approaching the CD33 surface. This proposed mechanism of steric occlusion is strongly supported by previous functional evidence obtained from patient-derived samples [29]. Specifically, competitive binding assays on PBMCs from chronic hepatitis B (CHB) patients demonstrated that 10C8 effectively outcompetes and displaces pre-bound HBV virions from the CD33 surface [29]. This competitive exclusion was further validated by FRET analysis [29], where 10C8 treatment significantly reduced the energy transfer efficiency between CD33 and HBV, consistent with the physical barrier created by the Fab-10C8 as observed in our 2:2 complex structure. Collectively, these quantitative SPR data and established competitive assays confirm that 10C8 possesses the potency required to sterically preclude receptor engagement by multivalent sialoglycan ligands like HBsAg [29].

Second, recent structural studies suggest that CD33 signaling requires ligand-induced clustering rather than simple dimerization [38]. Our AUC-SV-confirmed 2:2 complex adopts a rigid and compact geometry (Fig. 4) that imposes dual constraints: (1) it prevents further lateral assembly into higher-order clusters, and (2) it restricts the conformational flexibility required for ITIM accessibility and SHP-1/2 recruitment. By stabilizing this specific 2:2 arrangement, 10C8 effectively traps CD33 in a signaling-incompetent state, providing a mechanistic explanation for the observed restoration of immune cell activity, including enhanced antigen presentation and cytokine secretion [29].

Our structural findings integrate with recent biophysical models of full-length CD33 to clarify the disruption mechanism. Recent work has demonstrated that while IgC1 domains can form homodimers, the CD33 transmembrane (TM) domain is inherently monomeric, suggesting that signaling is triggered by multivalent ligand-induced receptor clustering rather than monomer-to-dimer transitions [38]. In HBV infection, the virus particle acts as a multivalent scaffold presenting high-density sialoglycans that cross-link CD33 molecules into large-scale clusters. The antibody-induced conformational constraints including the ~21° reduction in dimer angle and the steric bulk of bound Fabs physically prevent the CD33 ectodomains from rearranging into such higher-order assemblies, thereby exerting a dominant-negative effect on receptor organization.

Notably, recent studies have revealed that the inhibitory function of CD33 is not conserved across species. In particular, an earlier study demonstrated that human CD33 represses phagocytosis through SHP-dependent signaling, whereas murine CD33 lacks such activity due to differences in its cytoplasmic ITIM motifs [40]. These findings underscore the importance of structural and functional studies using human CD33, especially in the context of immune modulation and therapeutic targeting. Our structural data reinforce this perspective by delineating a potential Fab-induced ectodomain conformation that is uniquely poised to antagonize glycan-mediated inhibitory signaling in human immune cells, thereby providing a structural framework for CD33-targeted immunotherapy.

## Conclusion

In summary, this study provides the first high-resolution structural basis for antibody-mediated antagonism of CD33, a key inhibitory Siglec exploited by HBV to suppress host immunity. By resolving the CD33-ECD/Fab-10C8 complex, we show that antibody engagement stabilizes a compact dimeric conformation that sterically blocks viral glycan binding and restricts conformational plasticity required for inhibitory signaling. These findings establish a mechanistic framework for how targeted antibodies can relieve HBV-induced immunosuppression and highlight CD33 as a tractable checkpoint for therapeutic intervention in chronic hepatitis B.

## Supplementary Information

Additional file 1.

## Data Availability

All the data supporting the findings of this study are available within the paper, and Supplementary files. The atomic coordinates and structure factors of CD33-ECD/Fab-10C8 complex have been deposited in the Protein Data Bank (PDB) under ID code 9VL2.

## Abbreviations

CD33: Cluster of differentiation 33 (Siglec-3)
CDR: Complementarity-determining region
CHB: Chronic hepatitis B
ECD: Extracellular domain
Fab: Fragment antigen-binding
HBsAg: Hepatitis B surface antigen
HBV: Hepatitis B virus
ITIM: Immunoreceptor tyrosine-based inhibitory motif
SHP-1/2: Src homology 2 domain-containing phosphatase 1 and 2
SIGLEC: Sialic acid-binding immunoglobulin-like lectin
