# Supplementary material for: Crystal structure of the CD33/Fab-10C8 complex elucidates the mechanism of antibody antagonism in HBV-induced immunosuppression
Source: J Biomed Sci. 2026 May 29;33:56. doi: 10.1186/s12929-026-01248-9 (PMC13220637; doi:10.1186/s12929-026-01248-9)
Supplement: Supplementary file 1 — Additional file 1. [file 12929_2026_1248_MOESM1_ESM.docx]

| **Supplementary Table S1. X-ray diffraction data and refinement statistics of CD33-ECD/Fab-10C8** | | |
| --- | --- | --- |
| **Crystal** | **CD33-ECD/Fab-10C8** | |
| **Data collection statistics** | |  |
| Source | | NSRRC-TPS 05A |
| Wavelength (Å) | | 0.99984 |
| Space group | | P1 |
| Resolution (Å) | | 3.2 |
| Unit cell parameters | |  |
| *a*, *b*, *c* (Å)  α, β, γ (^o^) | | 71.4 / 71.2 / 86.8  95.9 / 94.2 / 111.2 |
| Redundancy of reflection | | 1.9 (1.8)^a^ [1.9]^f^ |
| Completeness (%), overall | | 89.7 (71.6) [86.6] |
| Wilson B factor (Å^2^) | | 75.2 |
| *I*/*σ* (*I*), overall | | 19.5 (6.4) [31.8] |
| *R*_merge_^b^ (%), overall | | 5.1 (12.7) [3.4] |
| *R_pim_* (%), overall | | 5.1 (12.7) [3.4] |
| *R_meas_* (%), overall | | 7.2 (17.9) [4.8] |
| CC_1/2_, overall | | 0.99 (0.97) [0.99] |
| **Refinement statistics** | |  |
| Resolution (Å) | | 28.4- 3.2 |
| *R*-factor^c^/*R*_free_^d^ (%) | | 20.5 (31.0) [17.3]/ 25.2 (37.2) [20.3] |
| Reflections used in refinement | | 23442 (1170) [1563] |
| Reflections used for R_free_ | | 2010 (106) [150] |
| Number of residues | | 1282 |
| Number of atoms | |  |
| Protein | | 9840 |
| N-acetylglucosamine | | 112 |
| *B*-factor (Å^2^) | |  |
| Overall/CD33/Fab | | 89.3/107.1/80.5 |
| N-acetylglucosamine | | 127.2 |
| RMSD bond lengths (Å) | | 0.002 |
| RMSD bond angles (°) | | 0.54 |
| Ramachandran favored (%) | | 91.1 |
| Ramachandran allowed (%) | | 8.6 |
| Ramachandran outliers (%) | | 0.3 |
| Rotamer outliers (%) | | 0.09 |
| Clashscore | | 7.4 |
| PDB ID^e^ | | 9VL2 |
| ^a^ Values in parentheses are for the highest-resolution shell.  ^b^ *R*_merge_=Σ\|*I*−<*I*>\|/Σ*I*, where *I* is the observed intensity and <*I*> is the average intensity from multiple observations of symmetry-related reflections.  ^c^ *R*=Σ\|*F*_obs_−*F*_calc_\|/Σ*F*_obs_, where *F*_obs_ and *F*_calc_ are the observed and calculated structure factor amplitudes, respectively.  ^d^ *R*_free_ was calculated with 10 % of the total number of reflections randomly omitted from the refinement.  ^e^ Protein data bank identifiers for co-ordinates.  ^f^ Values in brackets are for the low-resolution shell (28.4-8.6 Å). | | |

**Supplementary Table S2. The detail interaction between two CD33 molecules.**

| Hydrogen bond (Å) | CD33-1 | CD33-2 |
| --- | --- | --- |
| 2.83 | PHE 177[O] **(βC)** | ARG 190[NH2] |
| 3.32 | PHE 177[O] **(βC)** | PHE 177[N] **(βC)** |
| 3.34 | TRP 179[O] **(βC)** | TRP 179[N] **(βC)** |
| 3.08 | SER 181[O] **(βC)** | SER 181[N] **(βC)** |
| 2.96 | TRP 179[N] **(βC)** | PHE 177[O] **(βC)** |
| 3.28 | SER 181[N] **(βC)** | TRP 179[O] **(βC)** |
| 2.5 | GLN 213[NE2] | GLY 188[O] |


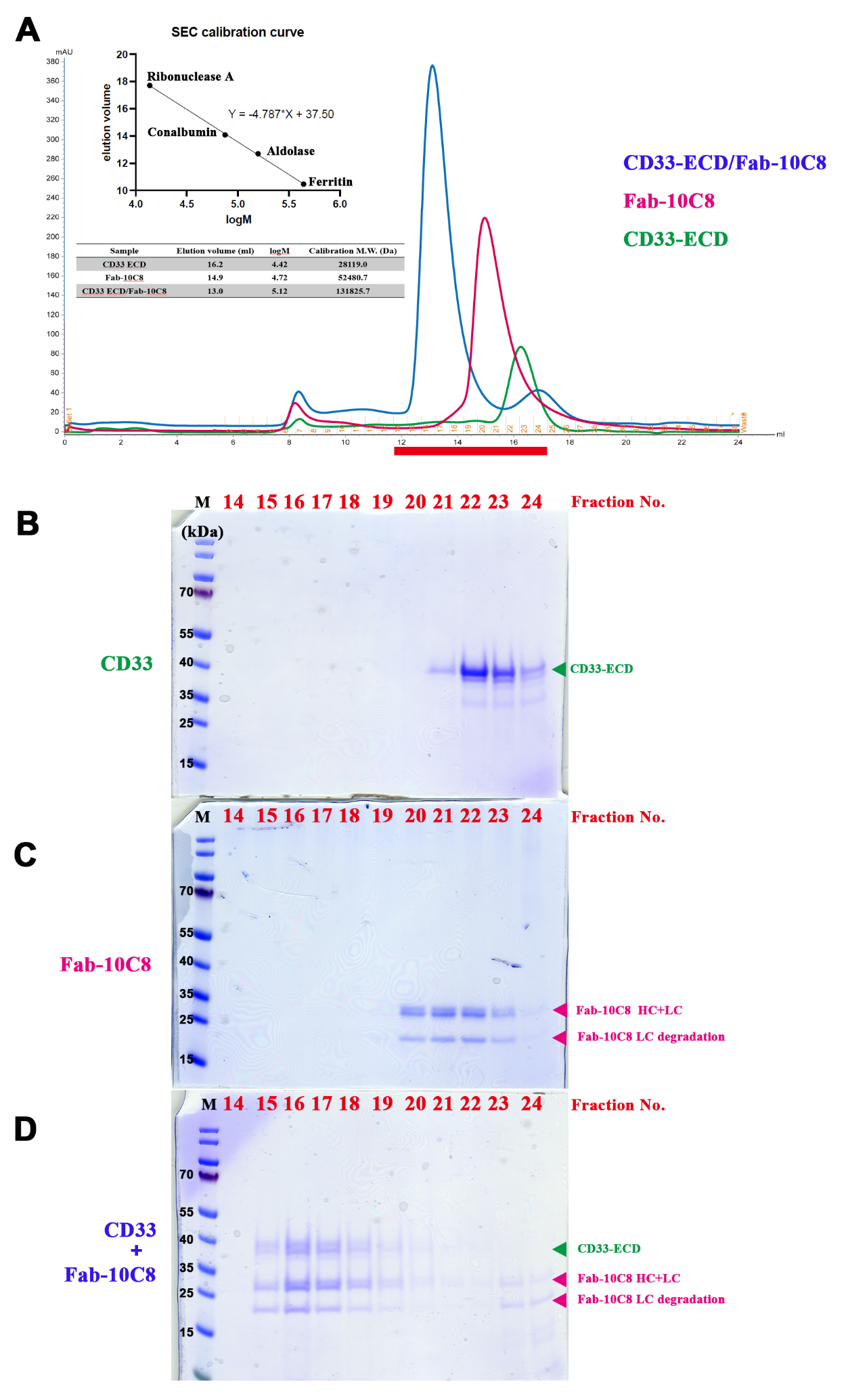


**Supplementary Figure S1. Biophysical characterization of CD33-ECD, Fab-10C8, and their complex.** (**A**) Size-exclusion chromatography (SEC) profiles of CD33-ECD (green), Fab-10C8 (magenta), and the CD33-ECD/Fab-10C8 complex (blue). The earlier elution of the mixture indicates stable complex formation. The inset shows the SEC calibration curve generated using molecular weight standards: ribonuclease A (13.7 kDa), conalbumin (75 kDa), aldolase (158 kDa), and ferritin (440 kDa). The table summarizes the calculated apparent molecular weights based on elution volumes. The red bar indicates the fractions (14-24) collected for subsequent SDS-PAGE analysis. (**B**-**D**) SDS-PAGE analysis of the fractions corresponding to CD33-ECD, Fab-10C8, and the co-purified complex. Molecular weight markers (M) and fraction numbers are indicated. Green and magenta arrowheads denote the bands for glycosylated CD33-ECD and Fab-10C8, respectively. Note that the Fab-10C8 heavy and light chains co-migrate as a single major band at ~25 kDa (labeled as Fab-10C8 HC+LC) due to their similar molecular weights. The lower minor band observed in panels (**C**) and (**D**) was confirmed by mass spectrometry to be a degradation product of the Fab fragment, rather than the intact light chain.


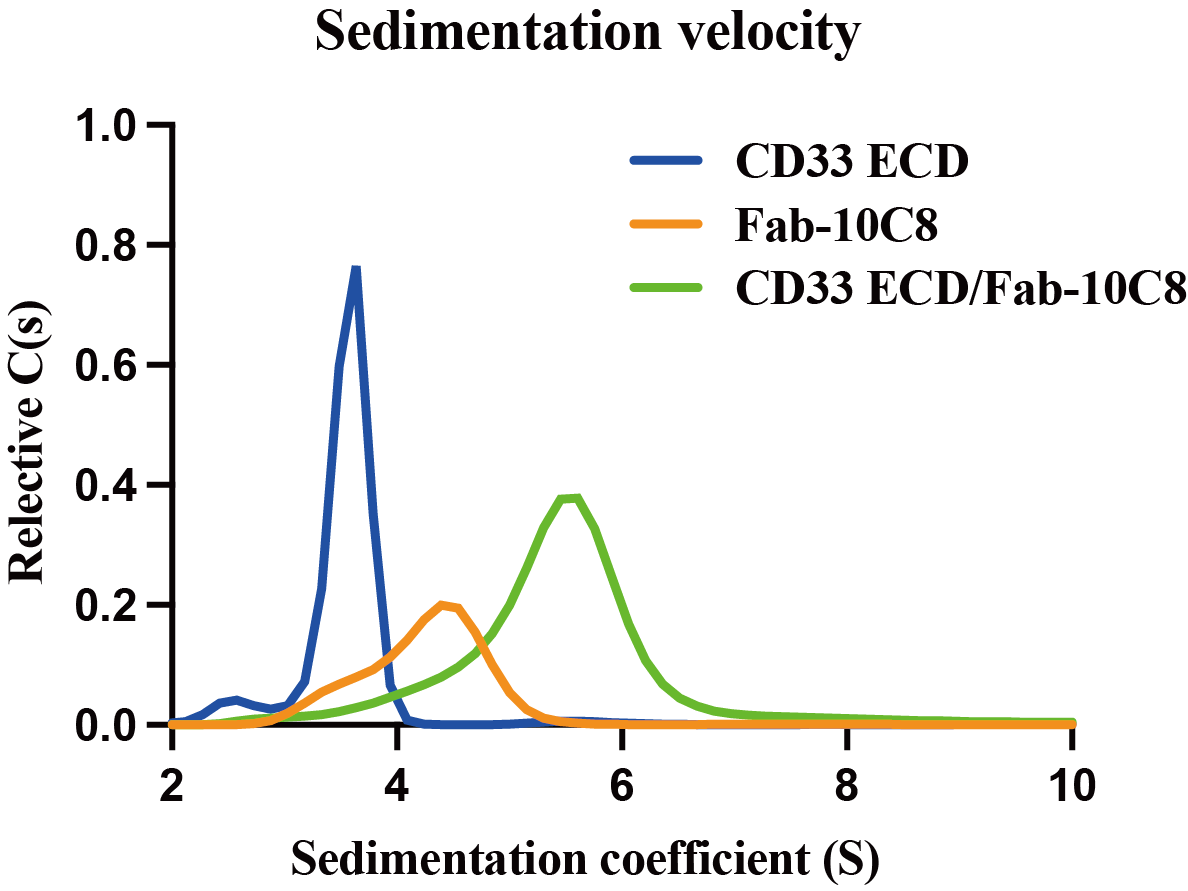


**Supplementary Figure S2.** **Sedimentation velocity analytical ultracentrifugation (SV-AUC) analysis of CD33 ECD, Fab-10C8, and their complex.** Continuous sedimentation coefficient distribution *c(s)* profiles of CD33 ECD (blue), Fab-10C8 (orange), and the CD33 ECD/Fab-10C8 complex (green) are shown as a function of sedimentation coefficient (*S*). CD33 ECD and Fab-10C8 exhibited molecular weights of 26.6 kDa and 48.7 kDa, with sedimentation peaks at approximately 3.7 S and 4.4 S, respectively. Upon mixing, the CD33 ECD/Fab-10C8 complex displayed a shifted peak at approximately 5.7 S, corresponding to a molecular weight of 157 kDa, consistent with a 2:2 stoichiometric assembly. All experiments were performed at 20°C in PBS.


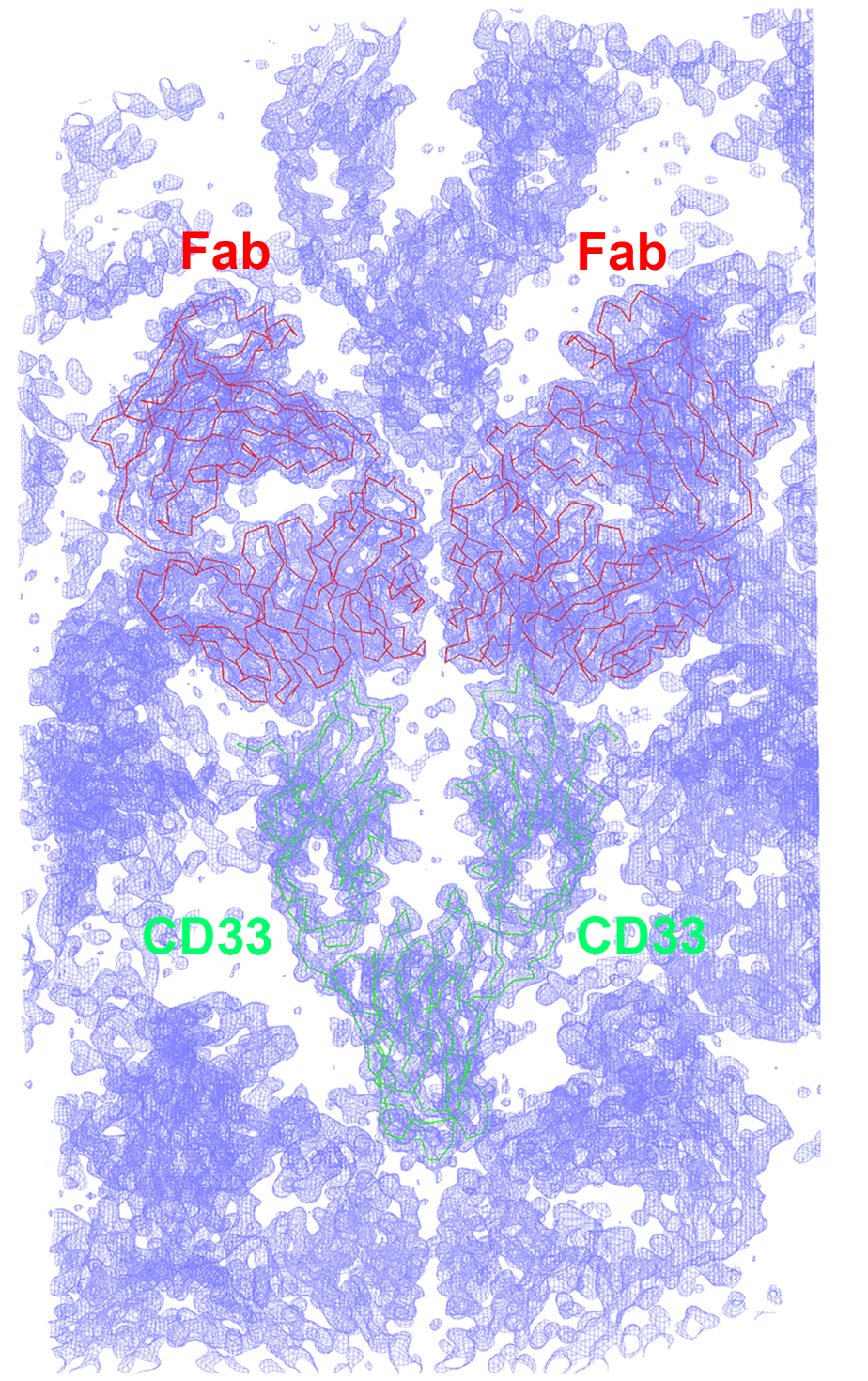


**Supplementary Figure S3. Electron density map of CD33-ECD/Fab-10C8 complex.** 2*Fo-Fc* electron density map (blue mesh, contoured at 1 σ) is shown with the refined model of the 2:2 CD33-ECD/Fab-10C8 complex. Fab-10C8 and CD33-ECD are shown in red and green, respectively.


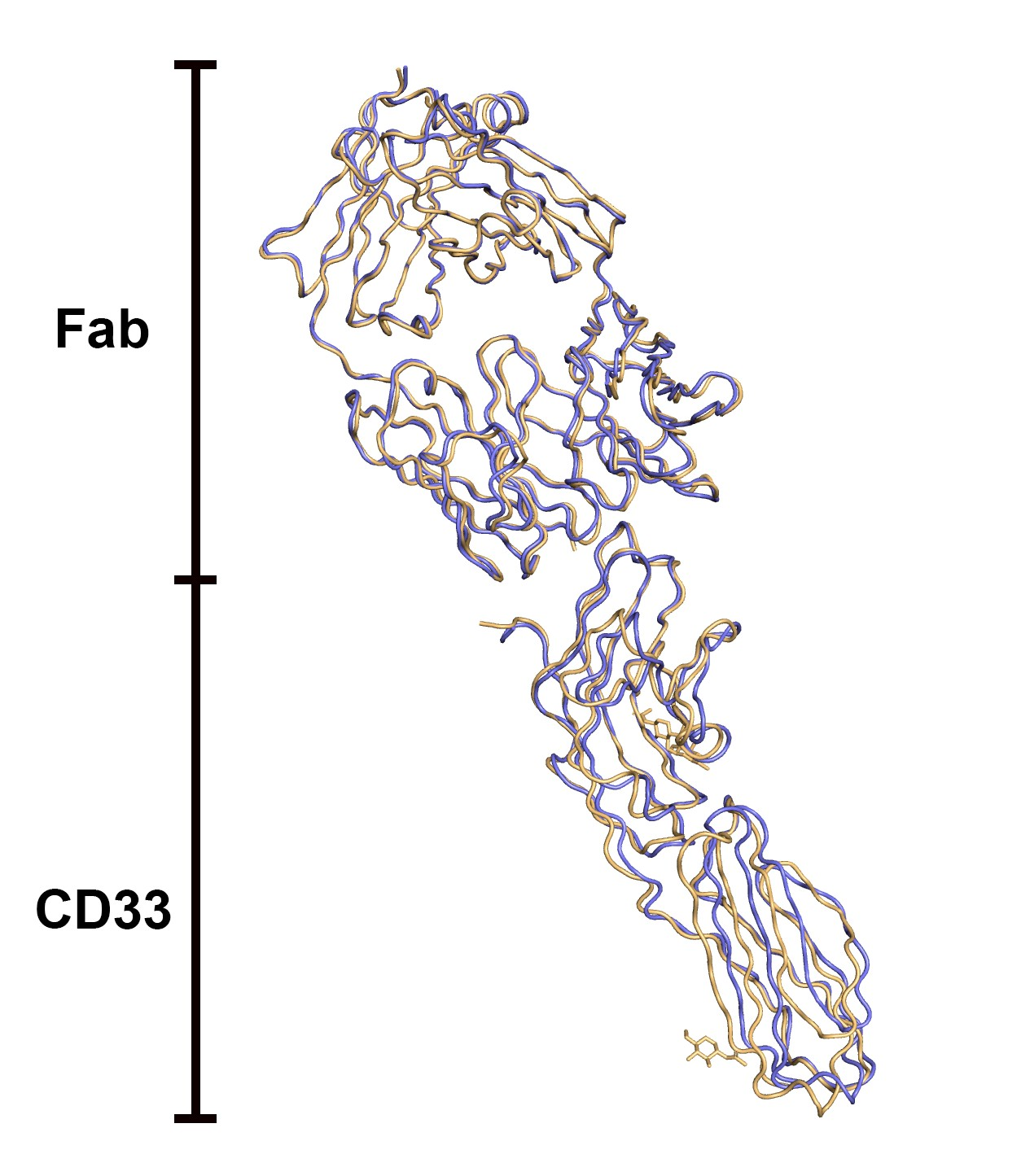


**Supplementary Figure S4. Structural superposition of two CD33-ECD/Fab-10C8 complexes.** Structural superposition of two CD33-Fab-10C8 complexes. The complexes are shown as backbone traces in orange and slate, demonstrating their high overall similarity and indicating minimal conformational variability between independent copies.


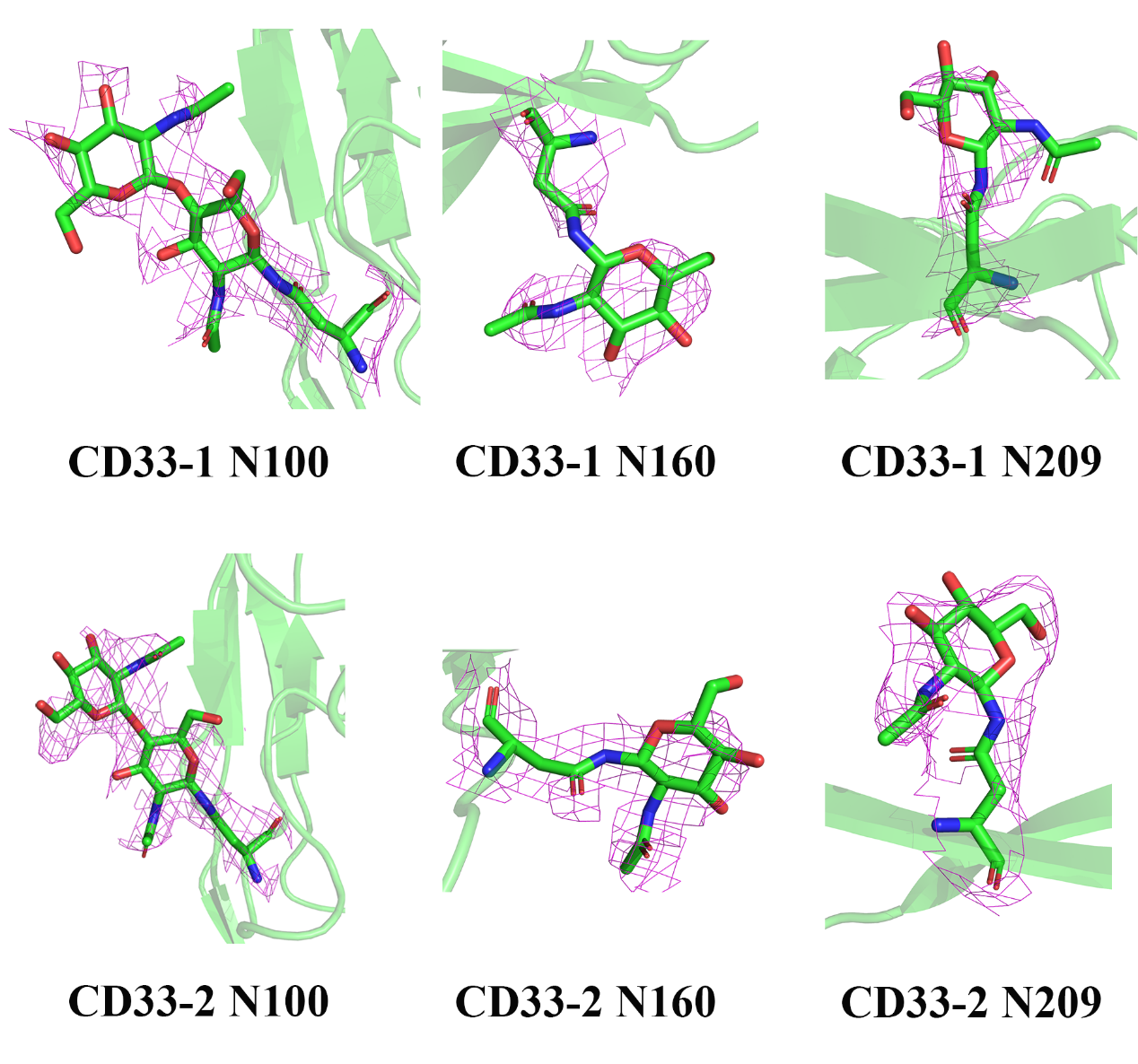


**Supplementary Figure S5. Electron density analysis of CD33 N-glycosylation sites.** The close-up views of the six reported N-glycosylation sites on CD33, with 2*Fo-Fc* electron density maps contoured at 1 σ. Clear density is observed at Asn100, Asn160 and Asn209.


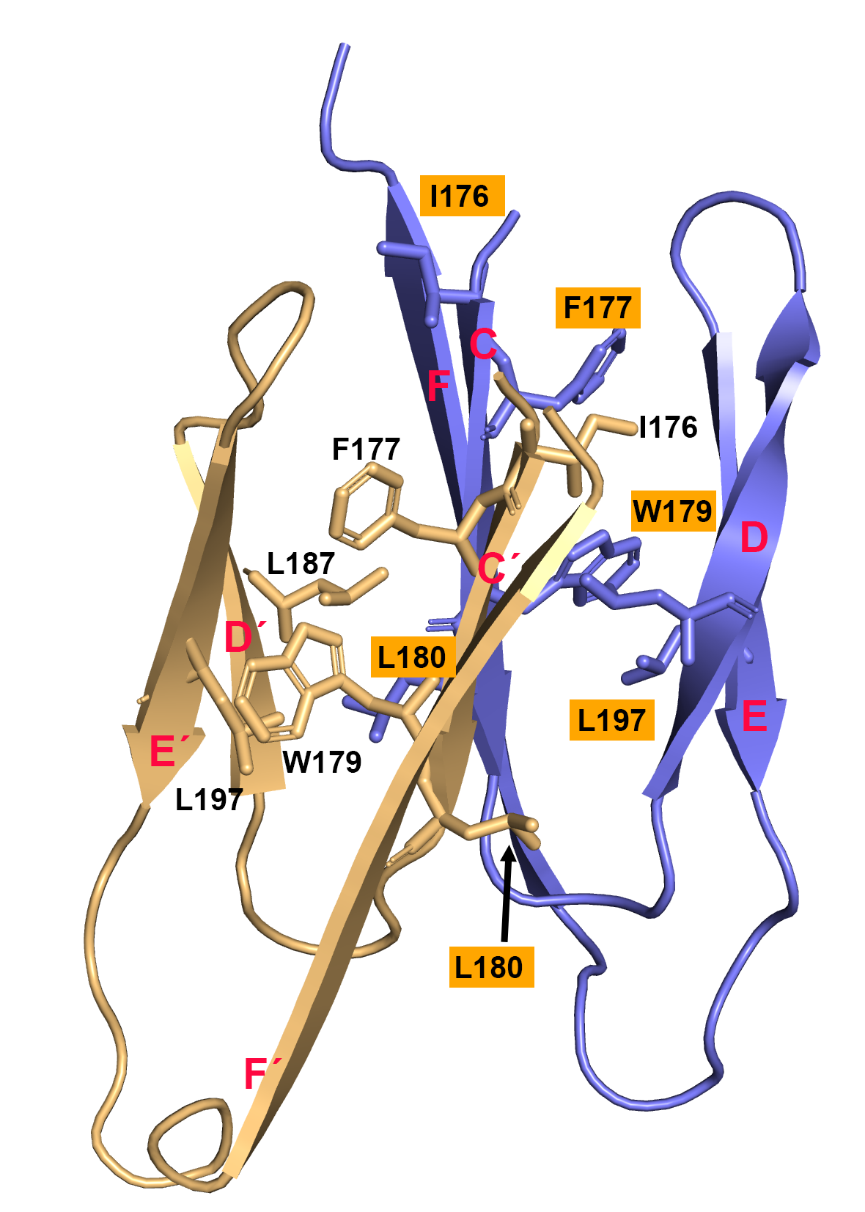


**Supplementary Figure S6. Hydrophobic network mediating CD33 C2 domain dimerization.** Close-up view of the CD33 C2-set dimer interface. Hydrophobic residues from opposing protomers interdigitate to stabilize the β-sheet pairing across the interface. Key residues include Ile176, Phe177, Trp179, Leu180, Leu187, and Leu197, which form an extensive hydrophobic cluster between the βC-βD-βE strands of one subunit (gold) and the βC′-βD′-βE′ strands of the other subunit (blue). This packing buries substantial surface area and provides the structural basis for stable C2 domain dimerization for CD33.


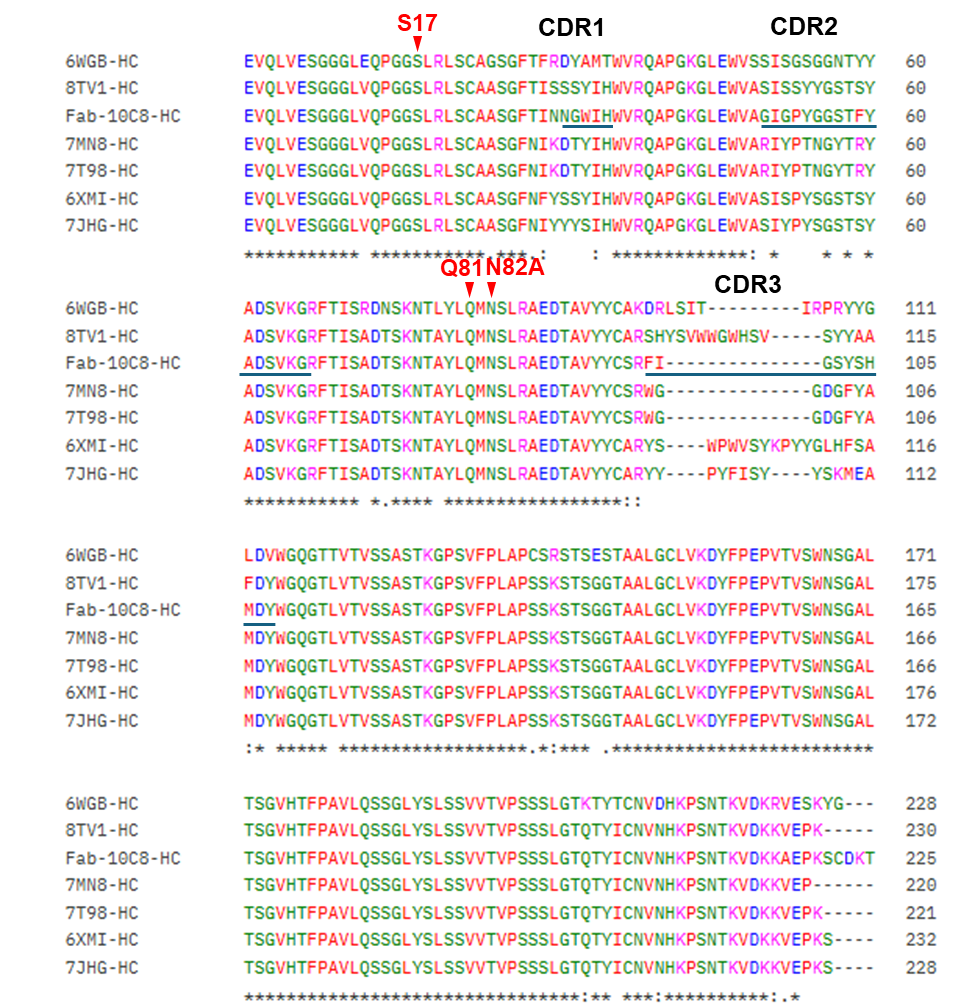


**Supplementary Figure S7. Sequence alignment of the Fab-10C8 heavy chain with representative structural homologs.** Multiple sequence alignment comparing the heavy chain (HC) of Fab-10C8 with several representative antibodies. The aligned homologs include PDB condes: 6WGB (Dupilumab), 8TV1 (EphA2 LBDCRD-bound FabS1C_L1), 7MN8 (Trastuzumab), 7T98 (engineered CYS-CYS Fab dimer), 6XMI (Fab4 bound to P22), and 7JHG (AMPK Fab-nanobody). Red arrowheads highlight the highly conserved framework residues Ser17, Gln81, and Asn82A, which are involved in the VH-VH inter-Fab interface observed in the CD33-ECD/Fab-10C8 complex. Blue lines denote the complementarity-determining regions (CDR1, CDR2, and CDR3) of Fab-10C8. Symbols below the sequence indicate the degree of conservation: asterisks (*) denote fully conserved residues, colons (:) denote conservation of strongly similar properties, and periods (.) denote conservation of weakly similar properties.


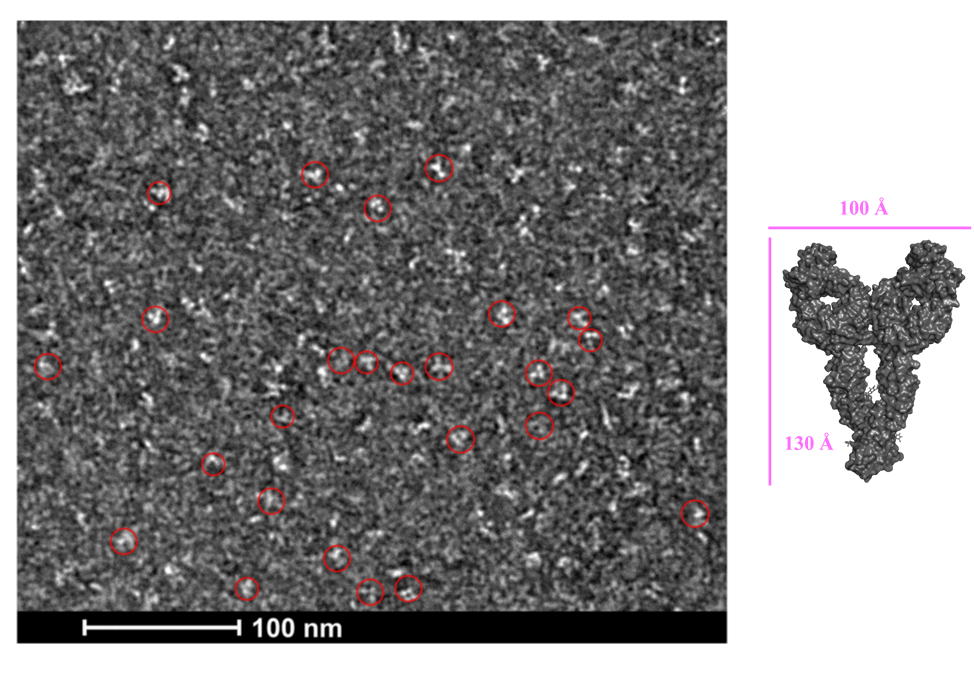


**Supplementary Figure S8. Negative-stain electron microscopy analysis of the CD33-ECD/Fab-10C8 complex in solution.** Representative negative-stain EM micrograph of the purified CD33-ECD/Fab-10C8 complex. The particles are highlighted with red circles, revealing a compact V-shaped architecture. The right panel displays the surface representation of the 2:2 complex from our crystal structure for comparison, with its dimensions indicated (approximately 130 Å in length and 100 Å in width). A scale bar of 100 nm is provided at the bottom.
